# Multiblock copolymers exhibiting spatio-temporal structure with autonomous viscosity oscillation

**DOI:** 10.1038/srep15792

**Published:** 2015-10-29

**Authors:** Michika Onoda, Takeshi Ueki, Mitsuhiro Shibayama, Ryo Yoshida

**Affiliations:** 1 Department of Materials Engineering, School of Engineering, The University of Tokyo, 7-3-1 Hongo, Bunkyo-ku, Tokyo 113-8656, Japan; 2 Institute for Solid State Physics, The University of Tokyo, 5-1-5 Kashiwano-ha, Kashiwa, Chiba 277-8581, Japan

## Abstract

Here we report an ABA triblock copolymer that can express microscopic autonomous formation and break-up of aggregates under constant condition to generate macroscopic viscoelastic self-oscillation of the solution. The ABA triblock copolymer is designed to have hydrophilic B segment and self-oscillating A segment at the both sides by RAFT copolymerization. In the A segment, a metal catalyst of chemical oscillatory reaction, i.e., the Belousov-Zhabotinsky (BZ) reaction, is introduced as a chemomechanical transducer to change the aggregation state of the polymer depending on the redox states. Time-resolved DLS measurements of the ABA triblock copolymer confirm the presence of a transitional network structure of micelle aggregations in the reduced state and a unimer structure in the oxidized state. This autonomous oscillation of a well-designed triblock copolymer enables dynamic biomimetic softmaterials with spatio-temporal structure.

Nanometer~sub-micron ordered spatial structures play a crucial role in living organisms; for example, the substrate-binding sites located inside enzyme proteins, selective ion transport channels within lipid bilayer membranes, compartmentalization regions formed by bilayer vesicles, etc. To mimic this scale of spatial structure, spontaneous self-assembly of amphiphilic block copolymers is frequently used^1^,^2^,^3^. A number of concepts to achieve valuable biomimetic materials from block copolymer, such as functional membrane^4^, vesicles^5^,^6^, nanoparticles^7^, and catalyst^8^, have been proposed.

Not only limited to the static spatial structure, microscopic temporal structures such as rhythm and oscillation are also known to influence macro dynamic processes in living organisms. These include energy transport and/or signal transduction via periodic formation of synaptic vesicles at nerve terminals^9^, periodic structural oscillation of fibroblast cells^10^, pulsation of cardiac muscle cells, amoeboid locomotion^11^,^12^,^13^,^14^,^15^, etc. In the straightforward movement of an amoeba, rhythmic sol-gel conversion occurs based on the hierarchical actin polymerization/depolymerization beneath the plasma membrane^13^,^14^,^15^. Protoplasma in the depolymerized sol state flow forward inside the cell and then reverse direction to fuse to gelled pseudopodium at the front of the cell. Concurrently, at the back of the cell, protoplasma in the form of polymerized gel immediately melts to a sol state and moves forward inside the cell again. Thus, the sol-gel transition is based on microscopic structure formation and break-up of the building blocks.

Block copolymers are often used as building blocks for forming self-assembled structures, as mentioned above. However, it is difficult to generate such an autonomous cyclic sol-gel transition from block copolymers because self-assembly forms under thermodynamically stable equilibrium state where the Gibbs free energy of the system is minimized. In spite to this difficulty, we recently succeeded in creating a unique spatio-temporal structure in non-equilibrium state of AB diblock copolymers under constant conditions by introducing a catalyst site of the Belousov-Zhabotinsky (BZ) reaction^16^, which is a well-known chemical oscillation reaction, into block copolymer architecture. The BZ reaction is often compared with the tricarboxylic acid (TCA) cycle, which is a key metabolic process that occurs in the living body, and is recognized as a chemical model for understanding several non-equilibrium phenomena in nature. The overall process consists of oxidation of an organic substrate, such as malonic acid (MA), by an oxidizing agent in the presence of a strong acid with the aid of a metal catalyst. During the reaction, the redox state of a metal catalyst, such as ruthenium bipyridine (Ru(bpy)_3_), undergoes a spontaneous rhythmic change.

With the redox change, the block copolymers show autonomous disintegration and reconstruction under constant conditions, although the random copolymers undergo expansion-contraction and swelling-deswelling in the case of linear polymer and gel, respectively^17^,^18^,^19^,^20^. By using the AB diblock copolmyer, we realized artificial oscillation phenomena, such as self-oscillating formation and break-up of micelles^21^, vesicles^24^, and the unique periodic structural transition of chemically cross-linked bilayer vesicles^25^, at the nanometer~sub-micron scale. Further, by designing architecture of block copolymer, it is expected that the oscillations are converted to higher-ordered structural changes leading to changes in macroscopic solution property.

In this paper, we demonstrate autonomous viscosity oscillation coupled with periodic aggregation and dissociation of an ABA triblock copolymer under constant conditions. The ABA triblock copolymer comprises poly(ethylene oxide) (PEO) as a hydrophilic central segment with a random copolymer of *N*-isopropylacrylamide (NIPAAm), methacrylamide with a Ru(bpy)_3_ side-chain as the metal catalyst for the BZ reaction, and *N*-(3-aminopropyl)methacrylamide (NAPMAm) as a precursor residue of the binding site of Ru(bpy)_3_ (Fig. 1(b)). The resultant triblock copolymers show low temperature unimolecular dissolution and high temperature micellization owing to the lower critical solution temperature (LCST)-type thermosensitivity of NIPAAm. The aggregation temperature strongly depends on the redox state of the Ru(bpy)_3_ incorporated into the self-oscillating segment because of hydrophilicity changes of the metal center (Fig. 1(a)). Therefore, the dilute triblock copolymer solution autonomously oscillates between unimolecular dissolution and aggregation with an appropriate amount of the BZ substrate under the bistable temperature at which the reduced block copolymer aggregates to form micelles while the oxidized polymer dissolves as individual polymer chains. By employing ABA triblock copolymer, micelle clusters are formed as transient precursors to the network structure. Then, during the oscillation, the microscopic structural changes are amplified to macroscopic viscosity oscillation. In this study, to analyze the spatio-temporal structure of the self-oscillating triblock copolymer, time-resolved dynamic light scattering (DLS) was used. From the comprehensive DLS analysis under various concentration conditions, the presence of micelle clusters was revealed as transient precursors to the network structure, which results in macroscopic viscosity oscillation with a large amplitude.

## Results and Discussion

### Self-assembly of the block copolymers in equilibrium

**Figure 1:**
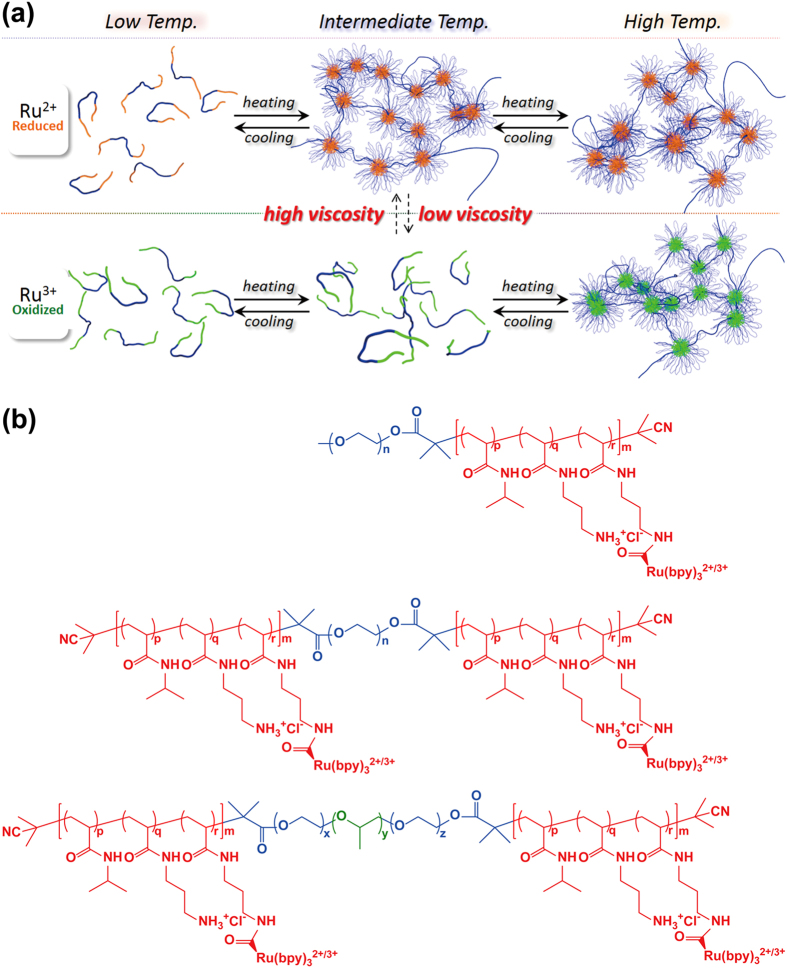
(**a**) Conceptual illustration of the periodic sol-gel transition based on autonomous oscillation of the ABA triblock copolymer. (**b**) Chemical structure of the self-oscillating AB diblock, ABA triblock and ABCBA pentablock copolymers.

A representative chemical structure of the self-oscillating ABA triblock copolymer is shown in Fig. 1(b). We first prepared a random copolymer of NIPAAm and NAPMAm and then attached the Ru(bpy)_3_ metal catalyst with an activated ester (i.e., Ru(bpy)_3_-NHS) to the amino side-chain of NAPMAm^26^,^27^. This strategy gives a significantly different chemical structure than that of the self-oscillating block copolymer prepared by one-step method using Ru(bpy)_3_ vinyl monomer in the earlier study^21^,^22^,^23^,^24^. The arrangement of NIPAAm and Ru(bpy)_3_ groups along the polymer chain is highly random because the electron densities of the propagation terminals of the polymers with NIPAAm and NAPMAm during polymerization process are comparable due to the similarity of the main-chain chemical structure; Ru(bpy)_3_-NHS is attached to the amino side-chain after random copolymerization.

**Figure 2 f2:**
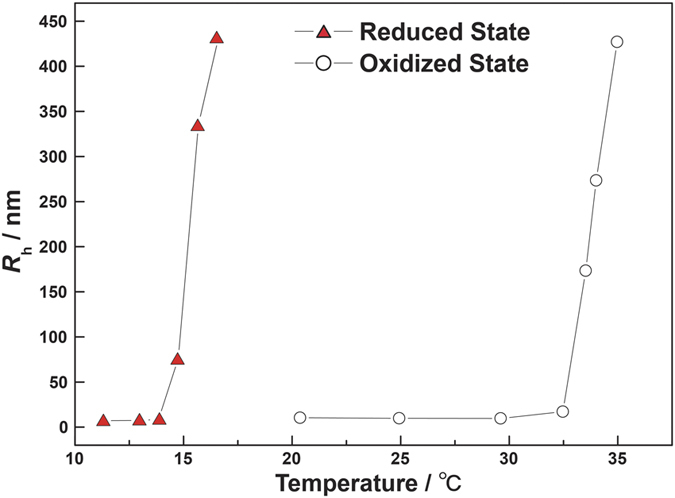
Relationship between hydrodynamic radius (*R*_h_) and temperature of reduced and oxidized ABA triblock copolymers (0.1 wt%). The redox states were achieved by adding 3 mM Ce_2_(SO_4_)_3_ and 1.0 M HNO_3_ (reduced state, Ru(bpy)_3_^2+^) and 3 mM Ce(SO_4_)_2_ and 1.0 M HNO_3_ (oxidized state, Ru(bpy)_3_^3+^), respectively.

Figure 2 compares the mean *R*_h_ of the ABA triblock copolymer (0.1 wt%) in reduced (Ru(bpy)_3_^2+^) and oxidized (Ru(bpy)_3_^3+^) states. During the heating process, the *R*_h_ significantly increases with increasing temperature because of the LCST nature of NIPAAm. This suggests that the triblock copolymer self-assembles into flower-like micelles with self-oscillating segment cores surrounded by well-solvated hydrophilic PEO shells looping-back to core at higher temperatures, being consistent with expectation^28^. The micellization temperature of the triblock copolymer is consistent with the previously reported phase transition temperature of PNIPAAm in block copolymers^26^,^27^,^28^,^29^,^30^,^31^,^32^,^33^,^34^,^35^,^36^,^37^,^38^. In oxidized state, the *R*_h_ at 20.3 °C below the lower critical micellization-type aggregation temperature (LCMT) is 10.4 nm, which is comparable with the sizes of the ABA single polymer chains with molecular weights of 225 kDa.

In both the reduced and oxidized states, increasing the temperature above LCMT results in an estimated *R*_h_ of >100 nm, which suggests network structures. In an amphiphilic diblock copolymer consisting of polybutadiene and PEO, vesicular morphology can occur when the weight fraction of the hydrophilic PEO is less than ~0.4^5^. Similar effects on the self-assembled structure with changes in the weight fraction of the block copolymer were also reported for a block copolymer with NIPAAm segments at higher temperature than LCMT^37^,^38^. Another possible explanation for the large aggregate is micellar aggregation of the ABA triblock copolymer. Zhou and co-workers recently reported the thermo-reversible aggregation behavior of a well-defined ABC terpolymer consisting of poly(ethylene-*alt*-propylene), PEO, and PNIPAAm as the hydrophobic, hydrophilic, and thermo-sensitive segments, respectively^39^. They observed a significant increase in *R*_h_ to >100 nm as the *M*_n_ of the PNIPAAm chain increased with constant molecular weights of the other two blocks; the large *R*_h_ was attributed to micellar aggregation around the PEO shell. Increasing the temperature above the LCST minimizes the contact of PNIPAAm chains with water through the formation of larger multi-micellar aggregates. We also confirmed the reversibility of the size changes of the scatters indicating unimer-micelle transitions in both the reduced and oxidized states.

### Autonomous oscillation of the block copolymers

**Figure 3 f3:**
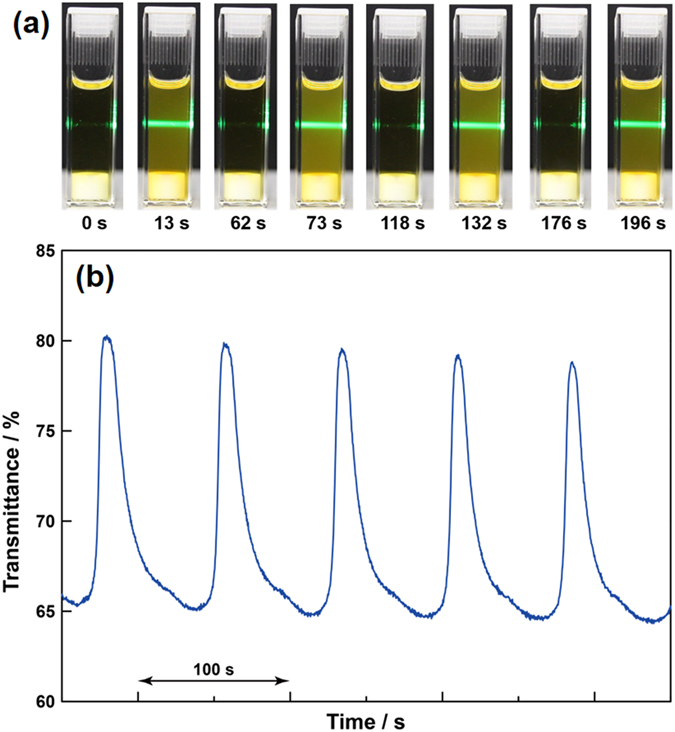
(**a**) Time course of the appearance of dilute ABA triblock copolymer (0.1 wt%) solution with an appropriate amount of BZ reaction substrate. The solution was magnetically stirred; the white object at the bottom of the solution is the stir bar. The sample was irradiated with green Ar laser (488 nm) from the left side of the sample to generate the Tyndall effect. (**b**) Self-oscillation of the transmittance of the dilute ABA triblock copolymer (0.1 wt%) solution at 20 °C. The concentration of the BZ substrates in the feed are as follows: [NaBrO_3_] = 0.2 M, [HNO_3_] = 0.3 M, and [MA] = 0.025 M.

Figure 3(a) shows photographs of the appearance of the ABA triblock copolymer solution (0.1 wt%) at room temperature with an appropriate amount of the substrate for the BZ reaction over time. The solution was irradiated with Ar laser (488 nm) from the left side of the sample. Periodic color changes between orange and light-green derived from the redox oscillation of Ru(bpy)_3_ are evident with periodic blinking of the laser light. When the sample is orange (reduced state), the Tyndall effect is clearly seen and indicates block copolymer aggregation, while no light scattering occurs in the light-green Ru(bpy)_3_ oxidized state. The transmittance of the ABA triblock copolymer solution at 570 nm under BZ reaction conditions also confirms that the turbidity of the solution oscillates autonomously (Fig. 3(b)), which suggests that block copolymer aggregation periodically changes as driven by the BZ reaction.

**Figure 4:**
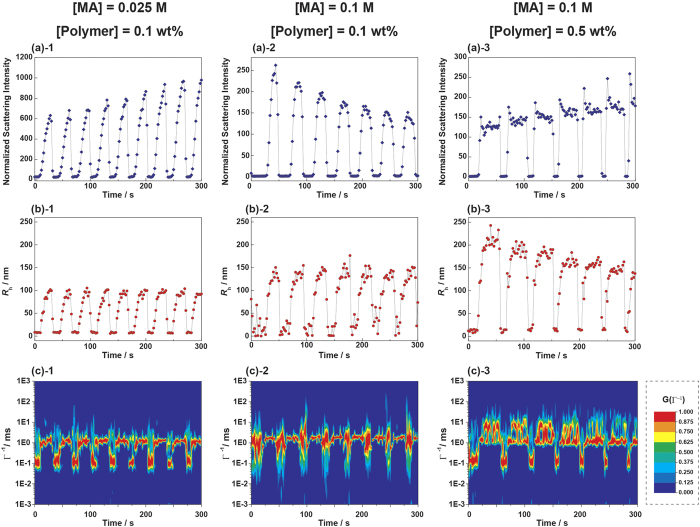
Oscillation behaviors of (a) normalized scattering intensity, (b) *R*_h_, and (c) the relaxation time distribution function, G(Γ^−1^), derived from CONTIN analysis during the BZ oscillation reaction for the ABA triblock copolymer.

To quantitatively investigate dynamic aggregation behavior, we monitored the size oscillation of particles in the block copolymer solution under various BZ substrate and polymer concentrations using time-resolved DLS. Figure 4(a–c) show the representative oscillation behaviors of the scattering intensity, *R*_h_, and the relaxation time distribution function (G(Γ^−1^)) indicated by RGB color, respectively, for the ABA triblock copolymer. As a result of measuring the average scattering intensity every 2 s, the periodic scattering intensity oscillation was clearly observed (Fig. 4(a)-1, 2, 3, [Fig f3]). From the oscillation waveform, it is clear that the decrease in scattering intensity that accompanies demicellization occurs much faster than the increase in intensity that accompanies micellization. This is explained by the characteristics of the BZ reaction where the process of autocatalytic oxidation or conversion of Ru(bpy)_3_^2+^ to Ru(bpy)_3_^3+^ occurs more rapidly than the opposite reduction process. Another possible explanation for the waveform may be a difference in the mechanism between the integration and reconstruction of aggregates, as discussed elsewhere^21^,^30^.

MA acts as a reductant during the reaction. Increasing the concentration of MA in the feed from 0.025 to 0.1 M while keeping the concentrations of the other BZ substrates and polymer solutions constant increases the length of the reduced period and size of the aggregates (Fig. 4(a)-1, 2, (b)-1, 2, [Fig f2], and Table S2). In the reduced period during oscillation, the decreased solubility of the self-oscillating segment is likely to result in aggregates. The higher MA concentration makes the block copolymer solution more reductive leading to the formation of aggregates being favored over unimolecular dissolution. Therefore, the reduction period is prolonged and the size of micelle is larger.

Furthermore, increasing the concentration of block copolymer results in the wave deformation to show a plateau in the reduced state, and the aggregates form larger particles through the reduction process (Fig. 4(a)-2, 3, (b)-2, 3, [Fig f3], and Table S2). This suggests that higher MA as well as triblock copolymer concentrations can promote the formation of a material-spanning network resulting in gelation or a highly viscous solution based on high molar mass aggregates when the solution is reduced. Incidentally, the waveform of normalized scattering intensity (Fig. 4(a)-1, 2, 3, [Fig f3]) and *R*_h_ (Fig. 4(b)-1, 2, 3, [Fig f3]) do not always correspond together. Although the exact reasons for the difference in waveforms between scattering intensity and *R*_h_ is still unclear, one possible explanation is described in terms of the consideration of form factor of aggregation structure in the Supplementary Information.

In an ABA triblock copolymer composed of B and A blocks with and without solvent selectivity, respectively, A chains basically need to overcome an energy penalty to dissociate and diffuse into the solvent matrix. The value of energy barrier depends on the incompatibility χ*N* between A block and solvent from a physically cross-linked transient network theory^40^, where χ is the Flory-Huggins interaction parameter and *N* is the degree of polymerization. From this consideration, self-assembled A domains in the solution will be subjected to network dissociation more easily when the molecular weight of the A segment reduces, leading to rapid formation/break-up network structure. On the other hand, when the molecular weight of the A segment increases, robust viscoelastic fluid (high viscosity solution or high storage elastic modulus gel at reduced state, transiently) might be organized, as expected. In these ways, the molecular weight of the blocks will be trade-off and an important factor which will affect oscillation of micellization/demicellization.

Comprehensive observations of the period of the block copolymer oscillation as a function of the concentrations of the BZ substrates confirm that the oscillation period decreases at higher initial concentrations of BZ substrates (NaBrO_3_, HNO_3_, and MA) in the feed (Figure S4). The oscillation period for the ABA triblock copolymer can be described using the following relationship:


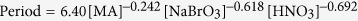


This tendency consistently agrees with other reports on BZ oscillation behavior, as judged by periodic color changes of the metal catalyst^41^,^42^. This result supports that the formation and break-up cycle of the ABA triblock copolymer is induced by the BZ oscillation reaction.

Another important aspect is the size distribution of the block copolymer aggregates during the oscillation. A series of Fig. 4(c) shows the result of time-resolved CONTIN analysis of the autocorrelation function obtained every 2 s. The time course of relaxation time distribution function, G(Γ^−1^), is indicated by RGB scale for relaxation time (Γ^−1^). Red indicates intense signals and blue indicates that no signal appears within the region of Γ^−1^. This analysis provides not only the average Γ^−1^ of the polymer in solution, but also the change in the distribution of these values over time. From the analysis, it can be seen that the distribution of Γ^−1^ is relatively broad and the signal intensity autonomously oscillates in all cases. The relaxation time distribution of the ABA triblock copolymer periodically changes more than one order of magnitude between the oxidized and reduced states as the BZ reaction occurs. In Fig. 4(c)-1, 2, 3, [Fig f3], intense signals observed at around Γ^−1^ = 0.1 and 1 ms correspond to the unimer and micelle, respectively. More interestingly, in addition to the oscillation of the faster relaxation mode derived from unimers and micelles, slower relaxation is evident around 10 ms when the concentrations of both the polymer and MA in the feed are the highest (Fig. 4(c)-3, [Fig f3]). This suggests that the formation of large aggregates of micelle clusters during oscillation as a possible precursor to the network structure, which has not been recognized from the discussion on the average Γ^−1^, could be detected. This one order slower Γ^−1^ is not observed in self-oscillating AB diblock copolymer with corresponding design (Figure S5).

**Figure 5:**
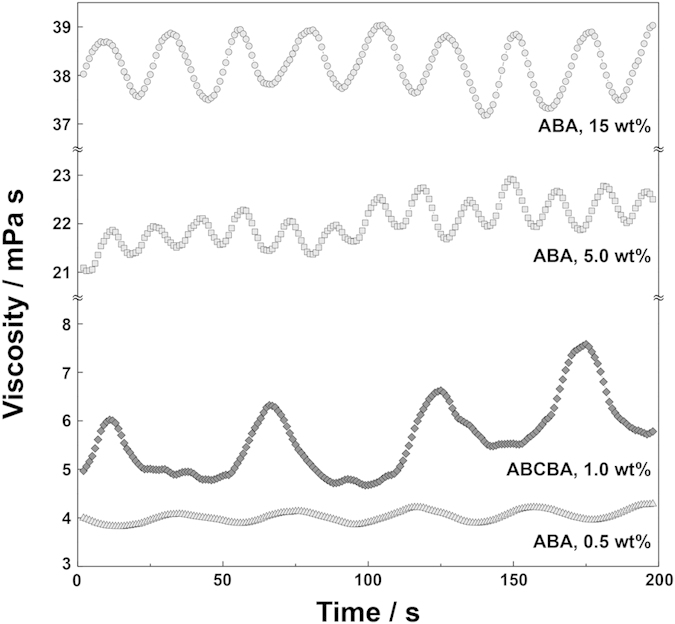
Viscosity oscillation driven by the periodic association and dissociation of the ABA triblock copolymers (0.5, 5.0, and 15 wt%) and an ABCBA pentablock copolymer (1.0 wt%) during the BZ reaction.

This result encouraged us to investigate viscosity oscillation driven by the BZ reaction under high concentrations of ABA triblock copolymer. We further studied the development of the microscopic oscillation mode in the aggregation state of the triblock copolymer to macroscopic viscoelastic oscillation. Viscosity measurements on the ABA triblock copolymer (polymer concentration = 0.5, 5, and 15 wt%) under BZ reaction conditions with higher MA concentrations were conducted (Fig. 5). Clearly, autonomous viscosity oscillation of the block copolymer solution is exhibited in all these cases. Increasing the concentration of ABA triblock copolymer results in monotonic increases in the oscillation amplitude as well as viscosity baseline. The oscillation amplitude of the 15 wt% ABA triblock copolymer solution is 1.5 mPa s with a viscosity baseline of 38 mPa s. This clearly indicates that the periodic changes in aggregate structure of the ABA triblock copolymer induce periodic changes of the molar mass of aggregation resulting in the macroscopic properties of the system. Here the amplitude of the viscosity is comparable to previous report^43^,^44^. One possible reason is that percolation of network structure was not achieved within 60 s, the period of the BZ reaction. In addition, under the BZ reaction oscillation condition, the redox state of incorporated Ru(bpy)_3_ into the random copolymer chain does not proceed to the complete oxidation or reduction states^41^. Aiming to increase the viscosity amplitude, we have synthesized ABCBA pentablock copolymer comprising self-oscillating A, hydrophilic B, and hydrophobic (insoluble) poly(propylene oxide) C blocks. The C block is introduced at the middle of the block copolymer architecture as a hydrophobic core to induce micelle formation during the oxidized state. By forming micelle with multi-arms, it is expected that network structure is easily formed at reduced state and hence viscosity amplitude is enhanced. As shown in Fig. 5, the viscosity oscillation of the ABCBA pentablock copolymer exhibits abound 2.0 mPa s amplitude with a baseline of around 5 mPa s. Although the polymer concentration is as low as 1.0 wt%, the largest amplitude was observed. This result supports that more effective network formation and break-up occurs by introducing hydrophobic center into the ABA block copolymer. Due to poor solubility of the ABCBA pentablock copolymer into aqueous solution, the solubility was found to be as low as 1 wt%. Although further optimization for controlling solubility would be necessary, the ABCBA pentablock copolymer designed to insert hydrophobic C block achieves the largest viscosity amplitude and shows the potential to realize sol-gel oscillation with self-oscillating multiblock copolymer system.

We previously reported autonomous viscosity oscillation coupled with rhythmic coordination and discoordination cycles of the terminal terpyridine ligand of a branched PEO with a Ru metal center depending on the redox states^43^. Also, sub-micron ordered well-defined self-oscillating microgel particles have been reported to induce autonomous viscosity oscillation^44^ with the dispersion and flocculation cycles under BZ reaction conditions. In this study, we displayed another example of induced viscosity oscillation from periodic changes of molecular aggregates. We are currently elucidating the mechanism of viscosity oscillation in detail. Among several strategies we have reported so far, designing self-oscillating block polymers as reported here would be more effective to increase the viscosity amplitude and realize sol-gel transition, according to further studies on multi-blocking design we are progressing now, which will be reported in the future. Such viscoelastic oscillation of the polymer solution can potentially enable its use as a functional fluid. Moreover, oscillating soft materials from straightforward synthetic polymer that express viscoelastic movement similar to amoeba motion can potentially act as model systems for simulation studies to achieve mechanical motion in living soft materials^45^.

In conclusion, we demonstrated autonomous viscosity oscillation induced by aggregation of ABA triblock copolymers without on–off switching. The target self-oscillating ABA triblock copolymer was successfully prepared by RAFT random copolymerization of NIPAAm and NAPMAm from PEO-base macro CTA followed by post-modification of Ru(bpy)_3_ activated ester to the amino residue of the NAPMAm side-chain. The ABA triblock copolymer exhibited a much wider range of bistable temperature between the reduced and oxidized states than previously reported self-oscillating AB diblock copolymers. Time-resolved DLS measurements for the oscillation at dilute block copolymer concentrations were carried out under various BZ substrate conditions. The comprehensive investigations confirmed the presence of the slower relaxation mode caused by micelle aggregation, which is a possible precursor of the polymer network, in addition to the faster relaxation modes of the unimer and micelle. The slower component appeared only under higher concentrations of MA (i.e., the reductant of the BZ reaction) and with higher concentrations of ABA triblock copolymer. In these conditions, remarkable viscosity oscillations were achieved.

## Methods

### Materials

*S*-1-Dodecyl-*S*’-(α,α’-dimethyl-α”-acetic acid)trithiocarbonate (CTA, purity >99%) was purchased from Trylead Corporation. Telechelic (–OH terminated) poly(ethylene oxide) (PEO), semi-telechelic PEO monomethyl ether, poly(ethylene oxide)_99_-*b*-poly(propylene oxide)_69_-*b*-poly(ethylene oxide)_99_ (F127), and 2,2′-azobisisobutyronitrile (AIBN) were purchased from Sigma-Aldrich. Anhydrous dichloromethane, 1,4-dioxane, and oxalyl chloride were purchased from Wako Chemicals. *N*-(3-Aminopropyl)methacrylamide (NAPMAm) was purchased from Polyscience. *N*-isopropylacrylamide (NIPAAm) was generously donated by the Kojin Corporation and purified via two times recrystallizations from a toluene/hexane (1:10 by weight) mixed solvent. AIBN was recrystallized from methanol prior to use. PEO and F127 was dried by azeotropic distillation using toluene prior to use. Bis(2,2′-bipyridine)(1-(4′-methyl-2,2′-bipyridine-4-carbonyloxy)-2,5-pyrrolidinedione)ruthenium(II) bis(hexafluorophosphate) (Ru(bpy)_3_-NHS) was synthesized according to the previously reported procedure^29^. All other chemical reagents were from Wako Chemicals and used as received unless otherwise noted.

### Synthesis of the Multiblock copolymer

Here, we describe a representative procedure for synthesis of the ABA triblock copolymer^23^,^30^. The triblock copolymer was synthesized following the procedure shown in Figure S2 in Supporting Information. First, the PEO-CTA macro-chain transfer agent was prepared as follows: Oxalyl chloride (0.74 mL, 8.6 × 10^−3^ mol) and CTA (0.63 g, 1.7 × 10^−3^ mol) were mixed in anhydrous dichloromethane (30 mL) under an Ar atmosphere and stirred at room temperature until gas evolution stopped (~2 h). Excess reagents were then removed under vacuum, and the residue was redissolved in anhydrous dichloromethane (100 mL) followed by the addition of telechelic PEO (6.0 g, 1.7 × 10^−4^ mol). The reaction was allowed to proceed for 24 h at room temperature after which the contents were reprecipitated two times using toluene as a good solvent and hexane as a poor solvent, respectively, and dried under vacuum at room temperature. The conversion for esterification of both end-groups was monitored by ^1^H-NMR spectroscopy. The reaction was carried out quantitatively. Yield: 5.5 g, 91%.

Next, a round-bottom flask was charged with PEO-CTA (4.67 g, 1.34 × 10^−4^ mol), NIPAAm (40.6 g, 3.6 × 10^−1^ mol), NAPMAm (2.0 g, 1.1 × 10^−2^ mmol) ([NIPAAm]/[NAPMAm] = 97/3 mol%), and methanol (solvent, 280 mL) and purged with Ar for 30 min. AIBN (25 mg, 1.5 × 10^−4^ mol) was separately dissolved in 10 mL of methanol (solvent) in another round-bottom flask and purged with Ar for 30 min at room temperature. All procedure was conducted under Ar atmosphere. An aliquot of the AIBN solution (3.5 mL, 5.3 × 10^−5^ mmol) was added to the monomer solution. RAFT random copolymerization was carried out at 60 °C for 48 h. The products were concentrated under reduced pressure and purified by three reprecipitations from toluene as a good solvent and hexane as a poor solvent.

Following the RAFT random copolymerization, the dodecyl trithiocarbonate residues derived from the CTA attached to the ABA polymer terminus were removed according to the following procedure: All obtained ABA triblock copolymer and appropriate amount of AIBN were dissolved in 290 mL of ethanol, and the solution was purged with Ar. The cleavage reaction was performed at 80 °C for 10 h under an Ar atmosphere. The product was concentrated under reduced pressure, and the resulting residue was purified by reprecipitation three times from toluene as a good solvent and hexane as a poor solvent and drying under vacuum at room temperature overnight. Subsequently, The ABA triblock copolymer (2.00 g, 1.7 × 10^−5^ mol) was dissolved in DMSO (30 mL) and added to a DMSO solution containing Ru(bpy)_3_-NHS (1.70 g, 1.7 × 10^−3^ mol). Triethylamine (7.8 × 10^−5^ mL, 5.6 × 10^−4^ mol) was added to the solution, and the solution was stirred for 48 h at room temperature. The obtained polymer was dialyzed against water and finally lyophilized for recovery. The structure and average molecular weight of the polymer were determined by a combination of ^1^H NMR spectroscopy and gel permeation chromatography (GPC) using dimethyl formamide (DMF) containing 1 mol L^−1^ LiBr as the carrier solvent (Figure S2). The total number average molecular weight (*M*_n_) of the triblock copolymer was calculated from the ratio of the integrated signal intensities of the PEO main chain and second block (δ = 3.7 ppm from NIPAAm, δ = 2.9–3.4 ppm from NAPMAm, and δ = 3.6 ppm from PEO). The NAPMAm composition was calculated using the ratio of the integrated signal intensities of NIPAAm and NAPMAm (Figure S3). The columns (Tosoh) for GPC analyses were calibrated using PEO standards. The characterization results for the polymers are summarized in Table S1. The *M*_*n*_ of the ABA polymer was 225 kDa. The AB diblock copolymer and the ABCBA pentablock copolymer were prepared similarly to the procedure mentioned above except that semi-telechelic PEO or F127 was used as a precursor instead of telechelic PEO.

### Measurements

All methods for the measurements of optical transmittance oscillation, dynamic light scattering measurements and rheological measurements are noted in supplementary information.

## Additional Information

**How to cite this article**: Onoda, M. *et al.* Multiblock copolymers exhibiting spatio-temporal structure with autonomous viscosity oscillation. *Sci. Rep.* **5**, 15792; doi: 10.1038/srep15792 (2015).

## Supplementary Material

Supplementary Information
