# Supplementary material for: Multiblock copolymers exhibiting spatio-temporal structure with autonomous viscosity oscillation
Source: Sci Rep. 2015 Oct 29;5:15792. doi: 10.1038/srep15792 (PMC4625142; doi:10.1038/srep15792)
Supplement: Supplementary Information [file srep15792-s1.pdf]

Supplementary Information

for

Multiblock copolymers exhibiting spatio-temporal structure with autonomous viscosity oscillation

Michika Onoda[a], Takeshi Ueki*[a‡], Mitsuhiro Shibayama*[b], and Ryo Yoshida*[a]

[a]Department of Materials Engineering, School of Engineering, The University of Tokyo, 7-3-1 Hongo, Bunkyo-ku, Tokyo 113-8656, Japan

[b]Institute for Solid State Physics, The University of Tokyo, 5-1-5 Kashiwano-ha, Kashiwa, Chiba 277-8581, Japan

[‡]Present address: National Institute for Materials Science, 1-1 Namiki, Tsukuba-city, Ibaraki 305-0044, Japan

E-mail: ueki@cross.t.u-tokyo.ac.jp, ryo@cross.t.u-tokyo.ac.jp, Tel & Fax: +81-3-5841-7112

Table of Contents

Measurements 2

Measurements of optical transmittance oscillation for polymer solutions 2

Dynamic light scattering measurements 2

Rheological measurements for polymer solutions 4

References 4

Figures 5

Figure S2 5

Figure S3 6

Figure S4 6

Figure S5 7

Figure S6 8

Tables 9

Table S1 9

Table S2 9

Measurements

Measurements of optical transmittance oscillation for polymer solutions

The polymer (0.1 wt%) was dissolved in an aqueous solution containing the reactants for the BZ reaction, i.e., 0.025 M malonic acid (MA), 0.2 M sodium bromate, and 0.3 M nitric acid. Under constant temperature and stirring conditions, the transmittance of the polymer solution at 570 nm (isosbestic point for reduced and oxidized states of $Ru(bpy)_3$) over time was recorded using a spectrophotometer (Shimadzu, Model UV-2500) equipped with an electronically controlled thermostated cell holder and magnetic stirrers.

Dynamic light scattering measurements

Sample solutions for DLS measurements were passed through 0.20 μm filters to eliminate dust prior to use. DLS measurements were performed on a DLS/SLS-5000 compact goniometer (ALV, Langen, Germany) coupled with an ALV photon correlator. A 22 mW He-Ne laser (Uniphase Co. Ltd., U.S.A.) was used as the light source. The wavelength of the light in vacuum was 632.8 nm. Although the laser power was relatively weak, the output photon-count rate was approximately 50 times higher than that of a conventional pinhole system; this was achieved by employing a set of static and dynamic enhancers (i.e., devices to enhance the photon counting rate) and a high quantum efficiency avalanche photodiode detection system. Experiments were performed at a range of temperatures (10–35 °C) with an accuracy of ±0.1 °C. The intensity autocorrelation functions, $g_2(q, t)$, were recorded at a scattering angle of 90° at each temperature for 30 s. Samples were equilibrated at a constant temperature for at least 30 min before data collection. When studying the BZ oscillation reaction, both autocorrelation function and time average scattering intensities were collected at a scattering angle of 90° for every 2 s. Experiments were performed at 20 °C with an accuracy of ±0.1 °C. Samples were equilibrated at a constant temperature for at least 30 min before data collection.

For solutions containing monodisperse particles, the electric field correlation function, $g_1(q, t)$, displays a single exponential decay, as follows:

$$g_1(q, t) = \exp(-\Gamma t) = \exp(-D_0 q^2 t) \qquad (1)$$

where q is the scattering vector ($q = (4\pi n/\lambda)\sin(\theta/2)$, where n is the refractive index of the solution, λ is the wavelength of light in vacuum, and θ is the scattering angle), Γ is the decay rate, and D_0 is the translational diffusion coefficient at the infinitely dilute limit. The recorded intensity correlation function, $g_2(q, t)$, was converted to $g_1(q, t)$ using the Siegert relation.[1] The hydrodynamic radius, R_h, can be estimated with knowledge of the solvent viscosity, η, using the Stokes-Einstein equation:

$R_h = (k_bT)/6\pi\eta D_0$ (2)

For solutions that contain polydisperse particles, $g_1(q, t)$ can be determined using the method of cumulants, as follows:[2]

$g_1(q, t) = A\exp(-\Gamma t)(1 + (1/2!)\mu_2 t^2 - (1/3!)\mu_3 t^3)$ (3)

where Γ is the mean decay rate and μ_2/Γ^2 characterizes the width of the distribution. In this work, the apparent R_h was calculated using Eq. (2) by substituting the $D = \Gamma/q^2$ of 0.1 wt% solutions for D_0.

The reciprocal of Γ is defined as relaxation time. The distribution of Γ^{-1} is expressed as relaxation time distribution function, $G(\Gamma^{-1})$, and it was examined by applying the inverse Laplace transformation to $g_1(q, t)$ using the well-established CONTIN program[3, 4] and by a sum of two exponentials.

In the dynamic light scattering measurements, scattering intensity, I is given by

$$I = NV^2P(qR_\mathrm{h}) \quad (1)$$

where R_h and V are the radius and the volume of the particle, respectively, and N is the number of particles in the irradiated volume. If all the unimer components in the polymer solution participate the aggregation, $P(qR_\mathrm{h})$ is written down as a form factor of the particle. When the aggregation is spheres, $P(qR_\mathrm{h})$ is given by

$$P(qR_\mathrm{h}) = \left(3\frac{\sin(q\mathrm{R_h}) - qR_\mathrm{h}cos\,(qR_\mathrm{h})}{(qR_\mathrm{h})^3}\right)^2 \quad (2)$$

where q is the wave vector, and q is given by

$$q = \frac{4\pi n_0}{\lambda_0}\sin\frac{\theta}{2} \quad (3)$$

where n_0 and λ_0 and θ are the refractive index of the solvent, wavelength of irradiated laser, scattered angle, respectively. Incidentally, when the aggregation structures is mono-modal spheres, V and N are given by

$$V = v \times n_{agg} = \frac{4\pi R_\mathrm{h}^3}{3} \quad (4)$$

$$N = \frac{N_{total}}{n_{agg}} = \frac{3vN_{total}}{4\pi R_\mathrm{h}^3} \quad (5)$$

where N_{total}, n_{agg}, v are the number of unimer, aggregation number, volume of micelle, respectively. Here, by using Eqs. (2), (4), (5), Eq (1) can rewrite as follows,

$$I = 12\pi vN_{total}R_\mathrm{h}^3\left(\frac{\sin(q\mathrm{R_h}) - qR_\mathrm{h}\cos\,(qR_\mathrm{h})}{(qR_\mathrm{h})^3}\right)^2 \quad (6)$$

By using Eq. (6), normalized scattering intensity can be expressed by a function of R_h (**Figure S1**). **Figure S1** strongly indicates the fact that the large R_h does not always give large scattering intensity.

There is a scattering intensity maximum against the size of the particle.

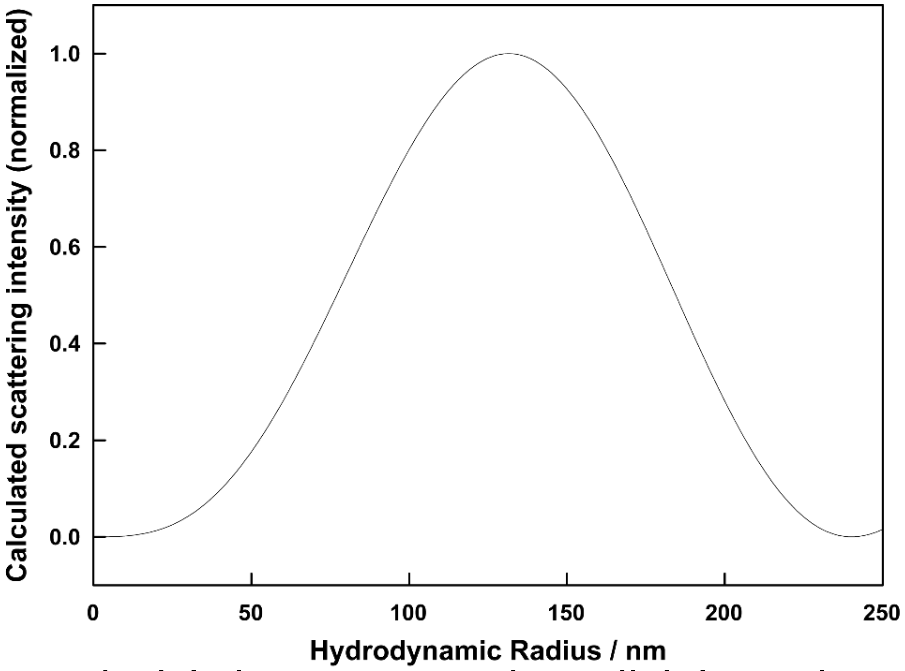

Figure S1 The calculated scattering intensity as a function of hydrodynamic radius.

Rheological measurements for polymer solutions

Rotational viscosity measurements were taken with an Anton Paar Physica MCR 301 rheometer using the cone plate geometry with 50 mm diameter plates (CP50-1). The viscosity was examined by applying a constant shear rate of 45 s^{-1}. The temperature was fixed at 20 °C.

References

1. Brown, W., Dynamic Light Scattering: The Method and Some Applications. *Clarendon Press, Oxford, England* (1993).

2. Koppel, D. E. Analysis of Macromolecular Polydispersity in Intensity Correlation Spectroscopy: The Method of Cumulants. *J. Chem. Phys.* **57**, 4814-4820 (1972).

3. Provencher, S. W. A Constrained Regularization Method for Inverting Data Represented by Linear Algebraic or Integral Equations. *Comput. Phys. Commun.* **27**, 213-227 (1982).

4. Provencher, S. W. CONTIN: A General Purpose Constrained Regularization Program for Inverting Noisy Linear Algebraic and Ingegral Equations. *Comput. Phys. Commun.* **27**, 229-242 (1982).

Figures

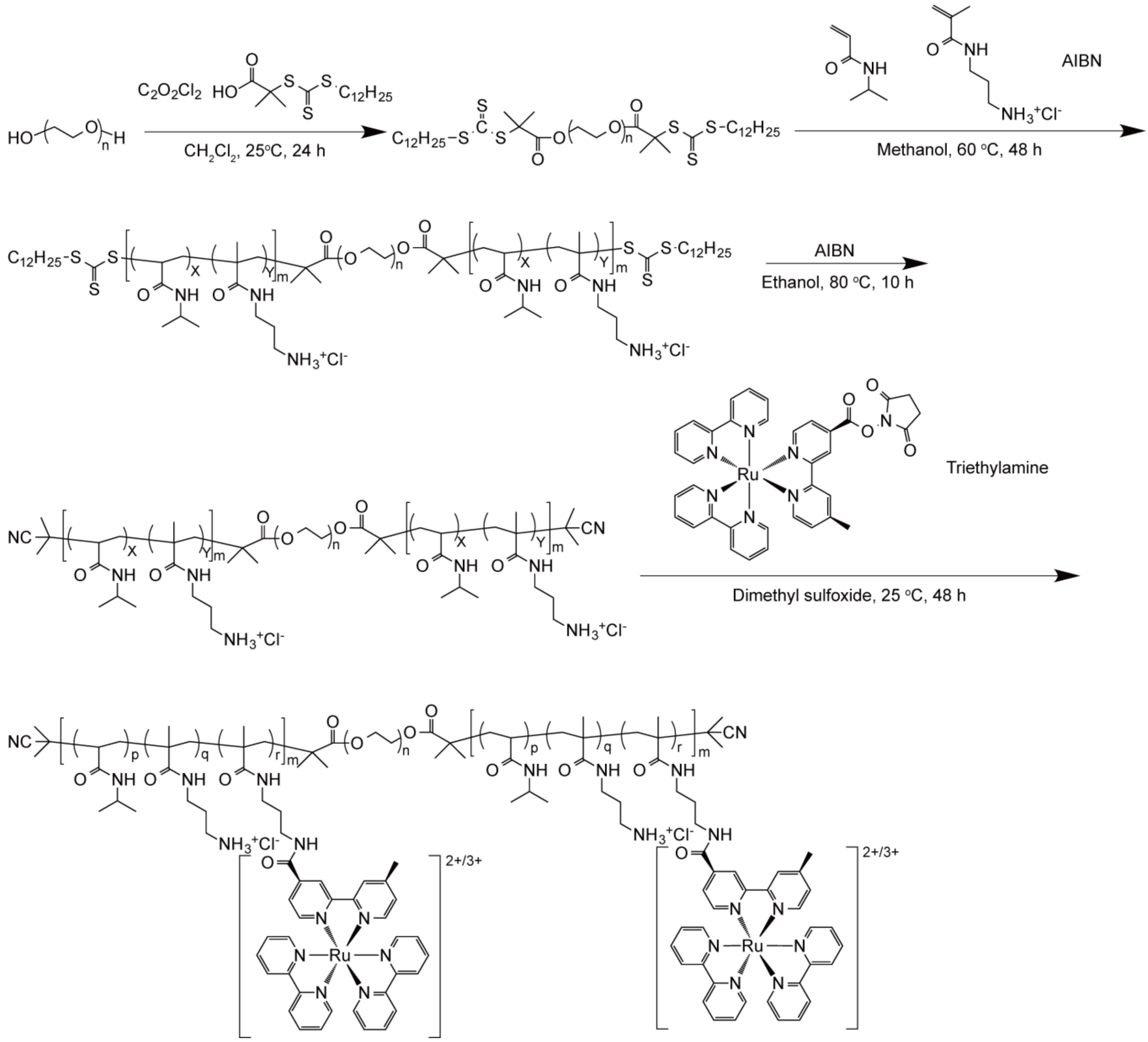

Figure S2. Synthetic procedure of the self-oscillating ABA triblock copolymer.

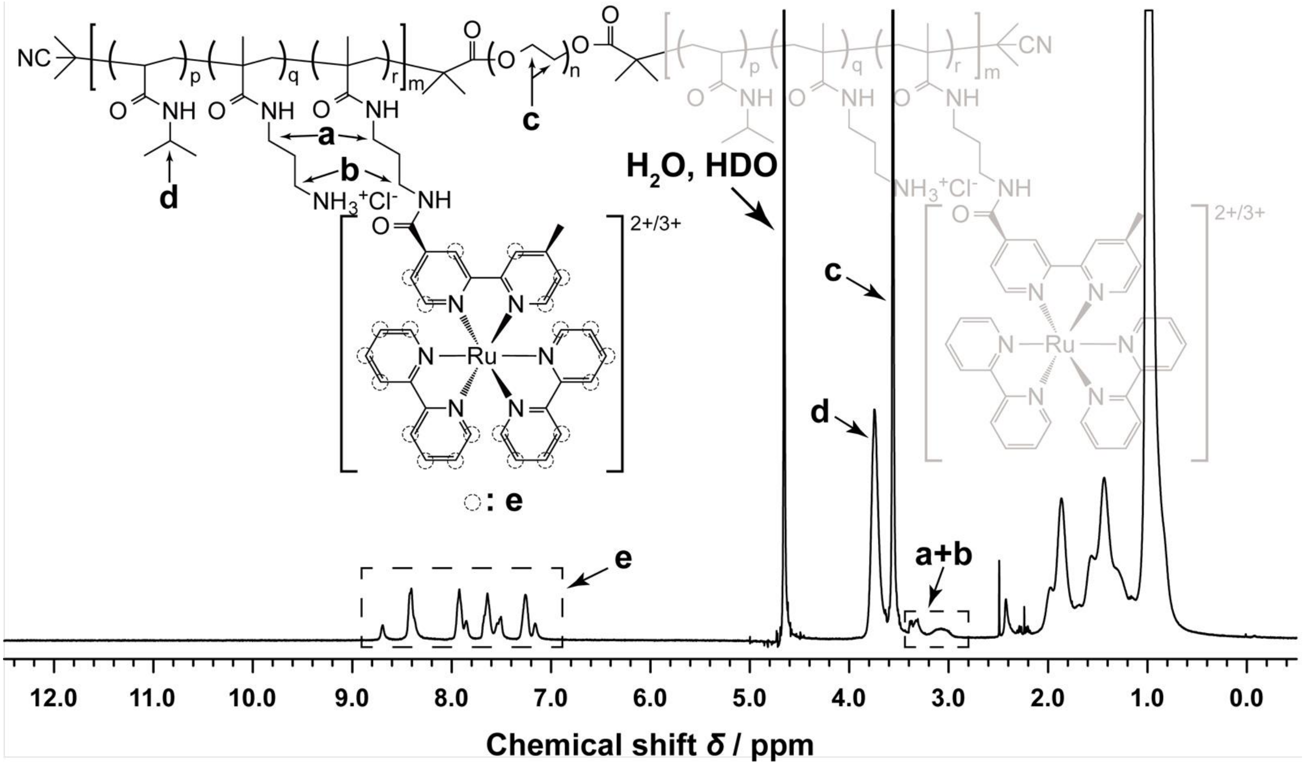

Figure S3. ^{1}H-NMR spectrum of the ABA triblock copolymer in D_2O.

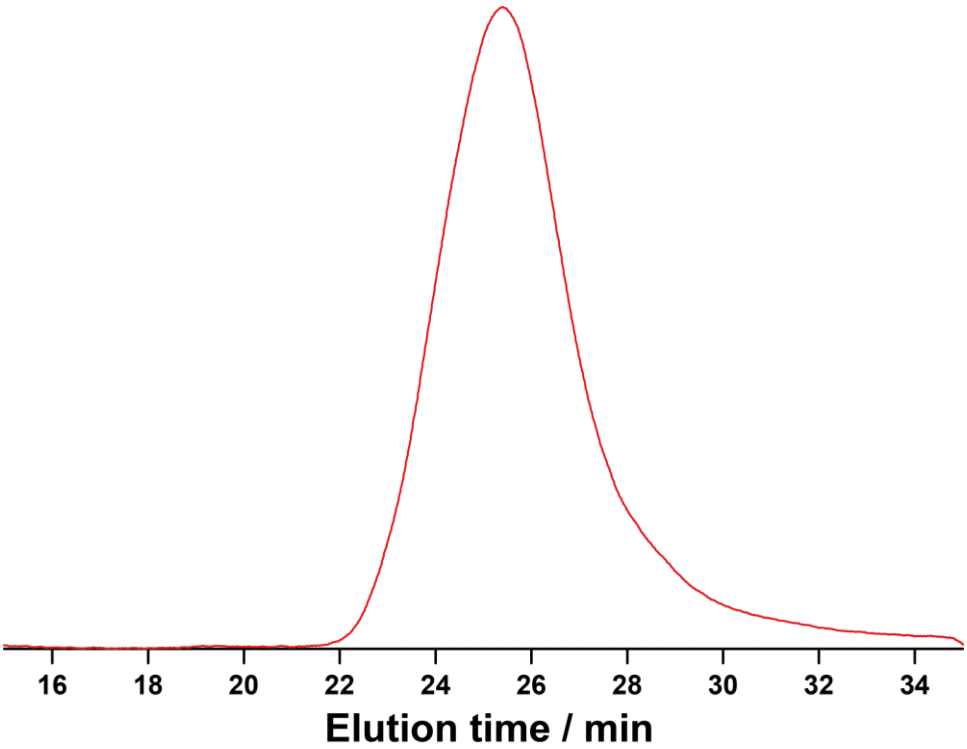

Figure S4. Size exclusion chromatography traces of the ABA triblock copolymer.

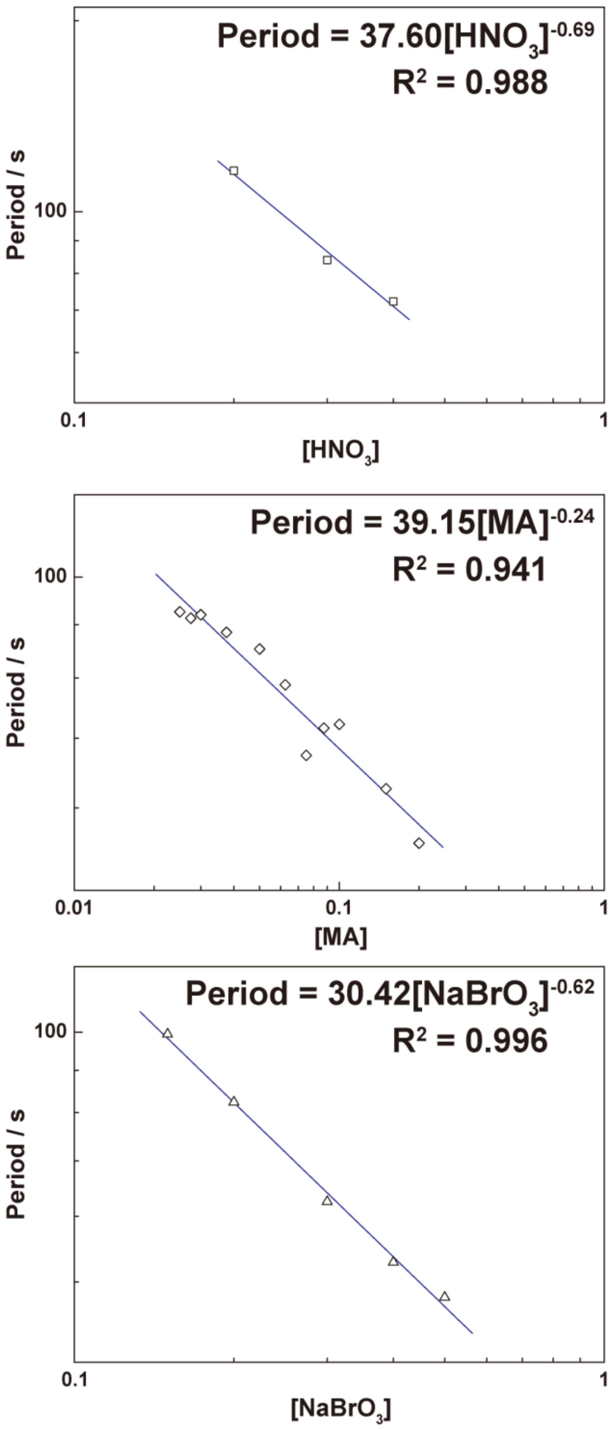

Figure S5. Oscillation period of the ABA triblock copolymer as a function of the concentration of (a) HNO_3, (b) MA, and (c) $NaBrO_3$.

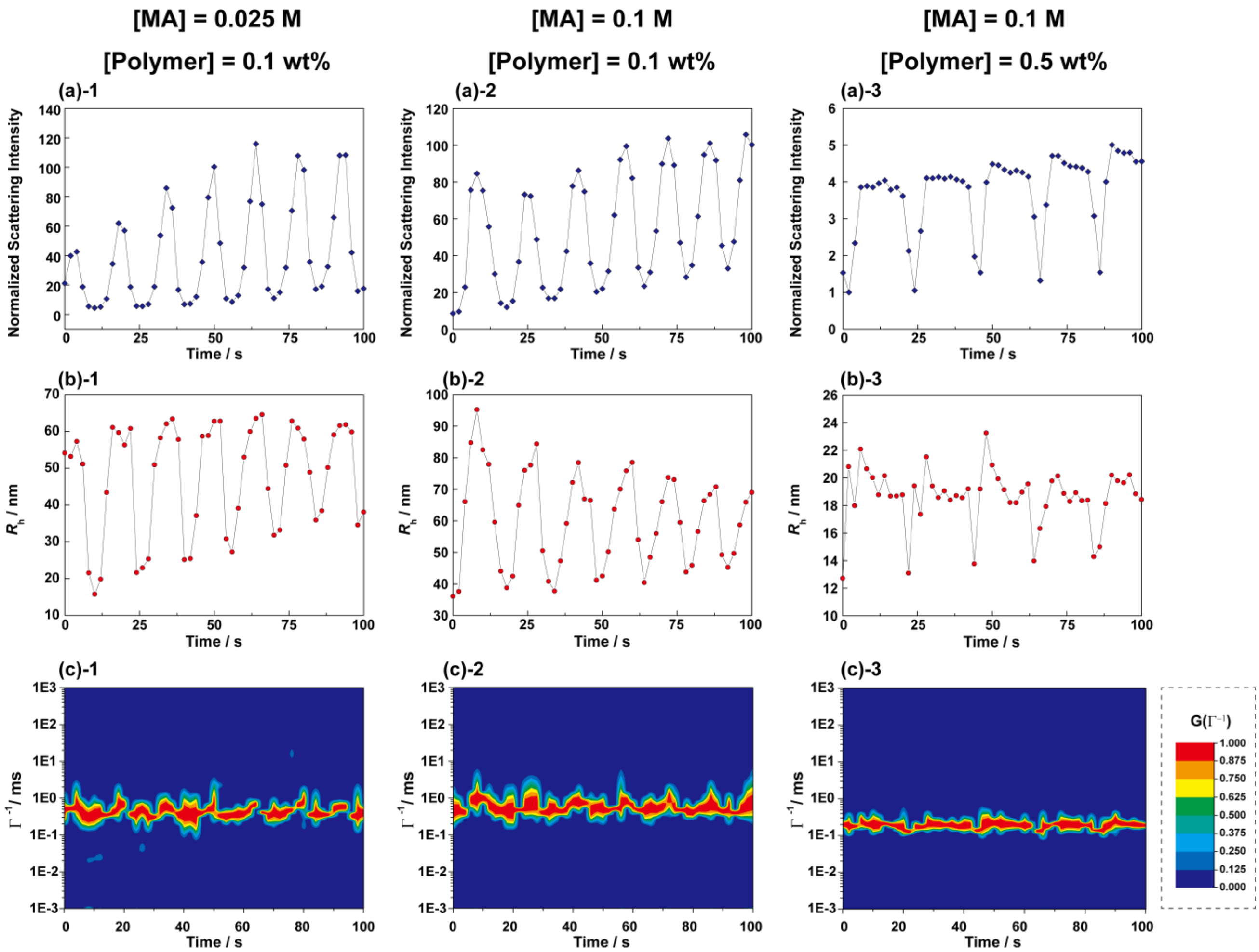

Figure S6. (a) Normalized scattering intensity (b) R_h and (c) time slice of the intensity distribution of the relaxation time of the translational diffusion from the CONTIN analysis during the BZ oscillation reaction for the corresponding AB diblock copolymer. Concentration condition for the oscillation in feed is same as indicated in **Table S1**.

Tables

Table S1. Summary of the ABA triblock copolymer.[a)]

Polymer	M_n / kDa	M_w/M_n	$x_{A\ seg.}$ [d)]
**PEO**	35 [b)]	1.11 [b)]	-
**ABA precursor** (before $Ru(bpy)_3NHS$ modification)<br>$P(NIP_{630}$-*r*-$NAP_{25})$-*b*-PEO_{795}-*b*-$P(NIP_{630}$-*r*-$NAP_{25})$	76-35-76 [c)]	1.78 [c)]	0.82
**ABA** (after $Ru(bpy)_3NHS$ modification)<br>$P(NIP_{630}$-*r*-NAP_4-*r*-$(Ru(bpy)_3NAP)_{22})$<br>-*b*-PEO_{795}-*b*-$P(NIP_{630}$-*r*-NAP_4-*r*-$(Ru(bpy)_3NAP)_{22})$	95-35-95 [c)]	1.77 [c)]	0.83
**AB** (after $Ru(bpy)_3NHS$ modification)<br>$P(NIP_{115}$-*r*-$NAP_{1.2}$-*r*-$(Ru(bpy)_3NAP_{1.1}))$-*b*-PEO_{113}	14-5.0	1.21	1.04
**ABCBA** (after $Ru(bpy)_3NHS$ modification)<br>$P(NIP_{125}$-*r*-NAP_3-*r*-$(Ru(bpy)_3NAP)_{10})$<br>-*b*-PEO_{99}-*b*-PPO_{69}-*b*-PEO_{99}-*b*-<br>$P(NIP_{125}$-*r*-NAP_3-*r*-$(Ru(bpy)_3NAP)_{10})$	27<br>-4.4-4.0-4.4-<br>27	-[e)]	0.52

a) Abbreviations of the monomer are as follows; NIP: *N*-isopropylacrylamide (NIPAAm), NAP: *N*-3-(aminopropyl)methacrylamide, PEO: poly(ethylene oxide), PPO: poly(propylene oxide), $Ru(bpy)_3NAP$: $Ru(bpy)_3$ moiety attached to amino group of NAPMAm via amide bond. Numbers after abbreviation of monomer indicate the number of repeating unit.

b) Estimated from GPC.

c) Estimated by combined with the results of GPC and ^{1}H-NMR.

d) Mole composition of each A segment in the block copolymer

e) Polydispersity of ABCBA was not measured because of the poor solubility to the carrier solvent.

Table S2. BZ substrates concentrations and results shown in Figure 4. The HNO_3 concentration for oscillation was fixed at 0.3 M.

Figure	[Polymer] / wt%	$[NaBrO_3]$ / M	[MA] / M	Period / s	R_h (ox) / nm	R_h (red) / nm
4(a),(b),(c)-1	0.1	0.2	0.025	34	6	101
4(a),(b),(c)-2	0.1	0.2	0.1	41	6	150
4(a),(b),(c)-3	0.5	0.2	0.1	45	8	208
